# S100A10 Accelerates Aerobic Glycolysis and Malignant Growth by Activating mTOR-Signaling Pathway in Gastric Cancer

**DOI:** 10.3389/fcell.2020.559486

**Published:** 2020-11-26

**Authors:** Yan Li, Xiao-Yu Li, Li-Xiang Li, Ru-Chen Zhou, Yinhe Sikong, Xiang Gu, Bi-Ying Jin, Bing Li, Yan-Qing Li, Xiu-Li Zuo

**Affiliations:** ^1^Department of Gastroenterology, Qilu Hospital, Cheloo College of Medicine, Shandong University, Jinan, China; ^2^Laboratory of Translational Gastroenterology, Qilu Hospital, Cheloo College of Medicine, Shandong University, Jinan, China; ^3^Robot Engineering Laboratory for Precise Diagnosis and Therapy of GI Tumor, Qilu Hospital, Cheloo College of Medicine, Shandong University, Jinan, China

**Keywords:** S100A10, gastric cancer, glycolysis, proliferation, mTOR

## Abstract

S100 calcium-binding protein A10 (S100A10) is crucially involved in the tumorigenesis of multiple malignant tumors. Reprogrammed glucose metabolism is emerging as a hallmark of various human cancers. However, the function of S100A10 in aerobic glycolysis is unclear. The expression of S100A10 was analyzed using the Oncomine database, Gene Expression Profiling Interactive Analysis (GEPIA), The Cancer Genome Atlas (TCGA), and the UALCAN cancer database. Prognostic analysis was performed using the Kaplan–Meier Plotter. The correlation between S100A10 and key glycolytic factors was assessed by GEPIA. The glycolysis level was examined by determining glucose consumption, lactate production, adenosine triphosphate production, cellular oxygen consumption rate, and extracellular acidification rate. Cell apoptosis was investigated by flow cytometry. Colony formation and BrdU assays were performed to detect cell proliferation. A subcutaneous xenograft mouse model was established to evaluate the effects of S100A10 *in vivo*. Gene Set Enrichment Analysis and western blotting were performed to explore the downstream signaling pathway. S100A10 was significantly upregulated in gastric cancer. Its expression was associated with poor survival. S100A10 increased glucose consumption, lactate production, and the switch from oxidative phosphorylation to aerobic glycolysis. S100A10 promoted malignant proliferation and suppressed cell apoptosis in gastric cancer. S100A10 activated the mTOR pathway by interacting with annexin A2 (ANXA2) to accelerate tumor glycolysis, resulting in tumor malignant progression. S100A10 contributed to aerobic glycolysis and accelerated malignant growth by modulating the Src/ANXA2/AKT/mTOR signaling pathway. Thus, S100A10 may have pivotal roles in gastric cancer.

## Introduction

Gastric cancer (GC) is the fifth most frequently diagnosed cancer and the third leading cause of cancer-related deaths worldwide (Bray et al., 2018). GC, a heterogeneous cancer, is traditionally classified into different histopathology subtypes based on histopathology using the World Health Organization (WHO) and Lauren classifications. GC is characterized into four subtypes (papillary, tubular, mucinous, and poorly cohesive) in the WHO scheme (Nagtegaal et al., 2020) and as intestinal, diffuse, and mixed subtypes in the Lauren scheme (Lauren, 1965). Additionally, a modified WHO classification (differentiated and undifferentiated) can be used to assess the risk of lymphatic metastasis (Gotoda, 2007).

These traditional classifications have not considered the molecular heterogeneity of GC and remain inadequate for individualized precision treatment (Wang Q. et al., 2019). Lei et al. (2013) compared the gene expression patterns of 248 gastric tumors and established a new molecular classification of GC. The authors identified three independent subtypes of GC: proliferative, metabolic, and mesenchymal. Patients with the proliferative subtype had shorter disease-free survival than patients with the other subtypes. Furthermore, the authors ascribed the increased sensitivity of the metabolic subtype to 5-fluorouracil treatment and the stem cell-like features of the mesenchymal subtype (Lei et al., 2013).

GC treatment and survival are largely dependent on the tumor stage. GC staging is divided into four stages according to the tumor node metastasis (TNM) classification system. The TNM staging system has fully considered the extent of the primary tumor (T), regional lymph nodes (N), and distant metastases (M) and provides a “stage grouping” based on T, N, and M (Amin et al., 2017). The TNM staging system is currently the major tool for clinicians to predict patient prognosis. However, GC patients with the same TNM stage often have diverse outcomes (Ahn et al., 2009), suggesting the existence of additional heterogonous factors influencing GC disease aggressiveness.

Uncontrolled proliferation and evasion of apoptosis are essential features of malignant tumors (Hanahan and Weinberg, 2011). Tumor cells rapidly proliferate, which requires more energy (DeBerardinis et al., 2008). Unlike normal cells, cancer cells are more likely to adopt aerobic glycolysis rather than mitochondrial oxidative phosphorylation. Aerobic glycolysis leads to high glucose consumption and lactate accumulation. These events allow rapid cell proliferation and tumor malignant growth (Warburg, 1956; Hanahan and Weinberg, 2011). Extremely low levels of glucose and high levels of lactate and glycolytic intermediates have been reported in GC tissues (Hirayama et al., 2009). Therefore, identification of novel molecular markers associated with aerobic glycolysis may allow the discovery of new efficient targets against GC.

S100 calcium-binding protein A10 (S100A10) is a member of the S100 protein family located in the plasma membrane. S100 proteins regulate a broad range of biological functions, such as phosphorylation regulation, maintenance of cell motility, and transduction pathway signaling (Santamaria-Kisiel et al., 2006). Recent studies have demonstrated that the S100A10 gene functions as a proto-oncogene. S100A10 is important in promoting tumor malignant growth in ovarian cancer, colorectal cancer, lung cancer, and pancreatic ductal carcinoma (Shang et al., 2013; Bydoun et al., 2018; Christensen et al., 2019). *In silico* analysis has revealed that S100 proteins are upregulated in GC (Liu et al., 2008). Typically, S100A10 functions as a scaffold protein connecting the two annexin A2 (ANXA2) molecules. The S100A10-ANXA2 complex (Erikson et al., 1984; Gerke and Weber, 1985a,b; Bharadwaj et al., 2013) is important in the progression of various cancers (Kumari and Malla, 2015; Christensen et al., 2019). Spijkers-Hagelstein et al. reported that high expression of S100A10 is necessary for successful phosphorylation of ANXA2 induced by Src kinase in mixed lineage leukemia (MLL)-rearrangements in infant acute lymphoblastic leukemia (Spijkers-Hagelstein et al., 2013). Moreover, phosphorylated ANXA2 is capable of activating the AKT/mammalian target of rapamycin (mTOR) signaling pathway to regulate cell proliferation (Kagawa et al., 2012; Zhang et al., 2018). Interestingly, the mTOR signaling pathway is crucial in aerobic glycolysis and cell proliferation (Wullschleger et al., 2006; Shackelford et al., 2009; Han et al., 2015). Thus, we hypothesized that S100A10 promotes glycolysis and proliferation by activating the mTOR signaling pathway.

To explore this hypothesis in the current study, we explored the function of S100A10 in GC aerobic glycolysis and investigated the underlying molecular mechanisms. To the best of our knowledge, this is the first evaluation of the biological functions and mechanisms of S100A10 involved in aerobic glycolysis in the progression of GC.

## Materials and Methods

### Analyses Involving Publicly Available Databases

#### Oncomine

Oncomine^1^ is a cancer microarray database and web-based data-mining platform (Rhodes et al., 2004). It was used to analyze the mRNA expression differences of S100A10 between tumors and corresponding normal tissues in various human cancers. The results are presented with a *P*-value of 0.01, fold change of 2, and gene ranking of all. The number in each colored cell indicates the number of datasets that met these thresholds. Cell color was determined by the gene rank. The color intensity (red or blue) is directly proportional to the significance level of upregulation or downregulation, respectively.

#### Gene Expression Profiling Interactive Analysis (GEPIA)

GEPIA^2^ is an interactive web application for gene expression analysis based on RNA sequencing data of 9736 tumors and 8587 normal samples from the Cancer Genome Atlas (TCGA) and the Genotype-Tissue Expression (GTEx) databases (Tang et al., 2017). We used GEPIA to determine the expression levels of S100A10 in distinct types of cancers. GEPIA was also used to determine the correlation between S100A10 expression and key genes during glycolysis in GC samples, including Solute carrier family 2 member 1 (SLC2A1, also termed GLUT1), SLC2A4 (GLUT4), Hexokinase 2 (HK2), Isocitrate dehydrogenase 1 (IDH1), Lactate dehydrogenase A (LDHA), 6-Phosphofructo-2-kinase/Fructose-2,6-biphosphatase 3 (PFKFB3), and Pyruvate kinase isoenzyme (PKM).

#### The Cancer Genome Atlas (TCGA)

To determine the expression of S100A10 in GC tissues, 375 GC and 32 normal tissue samples in which S100A10 was expressed were collected from the TCGA database^3^. All data were analyzed using R software (version 3.6.1).

#### University of Alabama Cancer Database (UALCAN)

UALCAN^4^ is an interactive and user-friendly web portal to perform in-depth analyses of TCGA gene expression data (Chandrashekar et al., 2017). We used UALCAN to assess the expression of S100A10 in the various stages of GC.

#### Kaplan–Meier Plotter

Kaplan–Meier plotter is an online database that includes gene expression data and clinical data. The plotter can analyze the expression of 54,675 genes and patient prognosis using 10,461 cancer samples (Szász et al., 2016; Pan et al., 2019). We utilized this database to explore the prognostic value of S100A10 expression in GC. Hazard ratios with 95% confidence intervals and log-rank *P-*values were also computed.

#### Gene Expression Omnibus (GEO)

The GSE35809 database was downloaded from the National Center for Biotechnology Information (NCBI) GEO database^5^. The GEO database contains genome-wide mRNA expression profiles of 70 primary gastric tumors from a cohort of Australian patients.

#### Gene Set Enrichment Analysis (GSEA)

GSEA is a computational analysis that is valuable in revealing the collective behavior of genes in various states of health and disease (Subramanian et al., 2005). In this study, TCGA data was analyzed by GSEA to determine biological processes enriched by S100A10. The samples were divided into a high S100A10 expression group (top 50%) and a low S100A10 expression group (bottom 50%). Gene set permutations were run 1000 times per analysis. The expression level of S100A10 was used as a phenotype label. The normalized enrichment score (NES), nominal *P*-value, and false discovery rate (FDR) were used to sort the pathways enriched in each phenotype.

### Cell Culture

Human GC cell lines that were used included AGS (well-differentiated gastric cancer cell line), HGC-27 (undifferentiated gastric cancer cell line), and MKN-45 (poorly differentiated gastric cancer cell line). They were purchased from the American Type Culture Collection (Manassas, VA, United States) and the Chinese Academy of Sciences (Shanghai, China). AGS was cultured in F12K medium supplemented with 10% fetal bovine serum (FBS, Gibco, Franklin Lakes, NJ, United States). The other two cell types were incubated in RPMI-1640 medium supplemented with 10% FBS. Cells were maintained in a humidified atmosphere of 5% CO_2_ at 37°C. All cell lines were authenticated by morphological observation and mycoplasma testing. Cells were grown for no more than 10 passages in total for any experiment.

### Plasmids, Small Interfering RNAs (siRNAs), and Cell Transfection

To construct an S100A10 overexpression plasmid, the full-length S100A10 coding sequence with a hexahistidine (6 × His) tag was amplified by PCR with forward (5′- CATGGTACCATGCATCATCACCATCACCATCCATCTCAAA TGGAACACG-3′) and reverse (5′-ATCTCGAGCTAA TGGTGATGGTGATGATGCTTCTTTCCCTTCTGCTTCATG-3′) primers. The underlined nucleotides represent the sequence encoding the 6 × His His tag fused in frame with S100A10. The amplified sequences were inserted into the pcDNA3.1 (+) (pcDNA3.1 (+)-S100A10) expression plasmid as described previously (Li Y. et al., 2019). The empty plasmid pcDNA3.1 (+) was used as a negative control. The 6 × His tag was added to help detect S100A10 protein expression.

The overexpression plasmids (1 μg/mL) were transiently transfected into GC cells using Lipofectamine 3000 (Invitrogen, Carlsbad, CA, United States). The transfection was performed 12 to 16 h after cells were seeded into 6- or 12-well plates with a cell density of 2 or 1 × 10^5^ cells per well, respectively.

S100A10 siRNA si-S100A10) and respective negative control (si-NC) were purchased from GenePharma (Shanghai, China). The sequences are si-S100A10 (*5′-*CCUGGACCAGUGUAG AGAUTT*-3′* and *5′-*AUCUCUACACUGGUCCAGGTT*-3′)*, si-NC (*5′-*UUCUCCGAACGU GUC ACGUTT*-3′* and *5′-*ACGUGACACGUUCGGAGAATT*-3′)*. The si-S100A10 (60 nM) was transfected into cells using Lipofectamine 3000 according to the manufacturer’s instructions.

### Lentivirus Infection

The S100A10-silencing lentivirus (short hairpin [sh]-S100A10), S100A10-overexpression lentivirus (OE-S100A10), and negative control lentivirus (sh-NC or OE-NC) were constructed by Genecopoeia (Rockville, MD, United States). GC cells were seeded in 6-well plates, grown overnight, and infected with lentivirus. The lentivirus titer was 1 × 10^8^ transduction units (TU)/mL. The infection rate of lentivirus was determined by fluorescence microscopy 72 h after infection. Puromycin (2 μg/mL) was added to the medium to kill the uninfected cells.

### RNA Extraction and Real-time Quantitative Real-time (RT-qPCR)

Total RNA was extracted using TRIzol reagent (Invitrogen). cDNA synthesis was performed using the ReverTra Ace^®^ qPCR RT Master Mix with gDNA Remover (Toyobo, Osaka, Japan) according to the manufacturer’s instructions. RT-qPCR was performed using SYBR^®^ Green Real-time PCR Master Mix (Toyobo) using the recommended thermal cycling settings of one initial cycle at 95°C for 60 s, followed by 40 cycles of 15 s at 95°C, 15 s at 60°C, and 45 s at 72°C. Gene expression was normalized to the expression of the housekeeping gene *glyceraldehyde 3-phosphate dehydrogenase* (*gapdh*). The primer sequences are listed in Supplementary Table S1.

### Western Blotting Analysis

Total protein was extracted from cells using RIPA Lysis Buffer (Solarbio Life Science, Beijing, China) and protein concentration was measured using the BCA Protein Assay Kit (Beyotime Biotechnology, Haimen, China). The proteins were separated by 10% SDS-PAGE and transferred to a polyvinylidene fluoride membrane. After blocking with 5% non-fat skim milk, the membrane was incubated with one of the following primary antibodies: 6 × His tag (1:1000, ab5000; Abcam, Cambridge, MA, United States), S100A10 (1:1000, 5529; Cell Signaling Technology, Danvers, MA, United States), Annexin A2 (1:1000, sc-28385; Santa Cruz Biotechnology), phosphor (p)-Annexin A2 (1:1000, sc-135753; Santa Cruz Biotechnology), Src (1:1000, ab109381; Abcam), p-Src (1:1000, ab185617; Abcam), AKT (1:1000, ab179463; Abcam), p-AKT (1:1000, ab192623; Abcam), mTOR (1:1000, ab32028; Abcam), p-mTOR (1:1000, ab109268; Abcam), 5′ adenosine monophosphate-activated protein kinase (AMPK, 1:1000, ab32047; Abcam), p-AMPK (1:1000, ab92701; Abcam), Glucose transporter 1 (GLUT1, (1:1000, ab115730; Abcam), LDHA (1:1000, ab101562; Abcam), PFKFB3 (1:1000, ab181861; Abcam), B-cell lymphoma 2 (Bcl-2, 1:1000, ab32124; Abcam), GAPDH (1:1000, ab9484; Abcam), and β-actin (1:1000, ab8227; Abcam). S100A10-overexpression efficiency was determined using 6×. His tag or S10010 antibody. S100A10 antibody was used to detect knockdown efficiency. Protein bands were quantified using Image J software (NIH, Bethesda, MD, United States) and expression levels were normalized to the internal reference. The experiments were repeated at least three times.

### Cell Apoptosis Assay

To examine the apoptotic effects of S100A10, GC cells transfected with siRNA or plasmids were grown in 6−well plates and incubated for 48 h. The apoptotic inducer tumor necrosis factor-related apoptosis-including ligand was added to the culture medium to a final concentration of 100 ng/mL for 24 h. The cells were collected by trypsin digestion in the absence of EDTA. Apoptosis was measured in the harvested cells using flow cytometry by the Annexin V-FITC Apoptosis Detection Kit (Bestbio, Shanghai, China) following the manufacturer’s protocol. Apoptosis rate (%) was calculated as the (total apoptosis/total cell number) × 100. The experiments were repeated at least three times.

### Cell Proliferation Assay

Cell proliferation was detected using a colony formation assay and Bromodeoxyuridine/5-bromo-2′-deoxyuridine (BrdU) assay. After transfection, 1000 cells were seeded in 6-well plates and grown for 2 weeks. The colonies were fixed, stained with 1% crystal violet, and counted to enumerate formative clones. The BrdU assay was performed as previously described (Li F. et al., 2019). Briefly, 48 h after transfection, GC cells were incubated with 10 μm BrdU (1:100, ab142567; Abcam) for 20 min and fixed with 4% paraformaldehyde (PFA). Cells were then treated with primary antibody to BrdU (1:1000, ab6326; Abcam) followed by a secondary antibody (1:1000, ab150165; Abcam). Finally, cell nuclei were stained with 4′, 6-diamidino-2-phenylindole (DAPI) and the cells were visualized by fluorescence microscopy. The experiments were repeated at least three times.

### Measurement of Glucose Consumption

Following transfection, cells were seeded into 6-well plates. Six hours later, the culture medium was replaced with complete medium and incubated for 48 h. Subsequently, the medium was collected to measure the glucose concentrations using a glucose assay kit (Shanghai Rongsheng Biotech Co., Ltd., Shanghai, China) according to the manufacturer’s instructions. Glucose consumption was calculated as the difference between the original glucose concentration in fresh medium and the measured glucose concentration in the collected medium. All results were normalized to the corresponding protein concentration values. The experiments were repeated at least three times.

### Measurement of Lactate Production

Lactate production in the culture supernatant was evaluated using a lactate assay kit (Jiancheng Bioengineering Institute, Nanjing, China) according to the manufacturer’s instructions. All results were normalized to the corresponding protein concentration values. The experiments were repeated at least three times.

### Measurement of ATP Production

The ATP production of GC cells was measured using an ATP assay kit (Nanjing Jiancheng Bioengineering Institute, Nanjing, China) according to the manufacturer’s instructions. All results were normalized to the corresponding protein concentration values. The experiments were repeated at least three times.

### Measurement of LDH Activity

LDH activity was determined using an LDH assay (Nanjing Jiancheng Bioengineering Institute) in accordance with the manufacturer’s instructions. The values were normalized to the protein levels. The experiments were repeated at least three times.

### Measurement of Cellular Oxygen Consumption Rate (OCR) and Extracellular Acidification Rate (ECAR)

OCR and ECAR were monitored using the XF96 Flux Analyzer (Seahorse Bioscience, North Billerica, MA, United States) following the manufacturer’s instructions. Briefly, 1 × 10^4^ cells were seeded into an XF96 well plate and allowed to adhere. To measure the OCR (pMoles/min), oligomycin, carbonylcyanide-4-(trifluoromethoxy) phenylhydrazone (FCCP), rotenone and antimycin A were sequentially added to each well. For ECAR, glucose, oligomycin, and glycolytic inhibitor 2-deoxyglucose (2-DG) were sequentially injected. The experiments were performed in triplicate.

### Tumor Xenograft Model

To determine the *in vivo* tumorigenicity, we established subcutaneous gastric cancer animal models. Firstly, we purchased S100A10-overexpression lentivirus (OE-S100A10) and negative control lentivirus (OE-NC) from Genecopoeia. The lentiviral overexpression system contained the enhanced green fluorescent protein (GFP) gene for tracking the infection efficiencies. HGC-27 cells were seeded in 6-well plates, grown overnight, and infected with lentivirus (OE-S100A10 or OE-NC). The infection rate of lentivirus was determined by fluorescence microscopy 72 h after infection. Green fluorescence of tagged GFP protein was monitored with excitation wavelength of 485 nm and emission wavelength of 525 nm in fluorescence microscopy. Successfully infected cells show green fluorescence under a fluorescence microscope. Puromycin was added to the medium to kill the uninfected cells, in order to ensure all cells were successfully infected with OE-S100A10 or OE-NC lentivirus. At last, fluorescence microscope was used to ensure that all cells were infected with lentivirus.

Then HGC-27 cells (1 × 10^7^) stably overexpressing S100A10 (OE-S100A10) or the corresponding control cells (OE-NC) were suspended in 200 μL PBS and subcutaneously injected into the right flank of 4-week-old BALB/c nude mice. After 3 days, the mice were randomly divided into rapamycin and PBS groups. Rapamycin was delivered by intra-peritoneal (i.p.) injection (100 μL) at 1.5 mg/kg/d body weight. The control group was administered 100 μL PBS. The tumor volumes were monitored every 5 days. The tumor volume was calculated as volume = (width^2^ × length) ÷ 2. After 35 days, the mice were sacrificed and the xenografts were removed for further study. The animal experiments were approved by the Shandong University Animal Care and Use Committee.

### Statistical Analyses

Statistical analyses were conducted using SPSS 25.0 (SPSS, Chicago, IL, United States) or GraphPad Prism 8.0 (GraphPad, La Jolla, CA, United States). Data from at least three independent experiments are expressed as mean ± standard deviation (SD). Differences between groups were analyzed using Student’s t-test (two-tailed). The correlation between genes was analyzed using Pearson’s correlation. The level of significance was determined with cut-offs at ^∗^*P* < 0.05, ^∗∗^*P* < 0.01, and ^∗∗∗^*P* < 0.001.

## Results

### S100A10 Is Upregulated in Various Human Cancers

**FIGURE 1:**
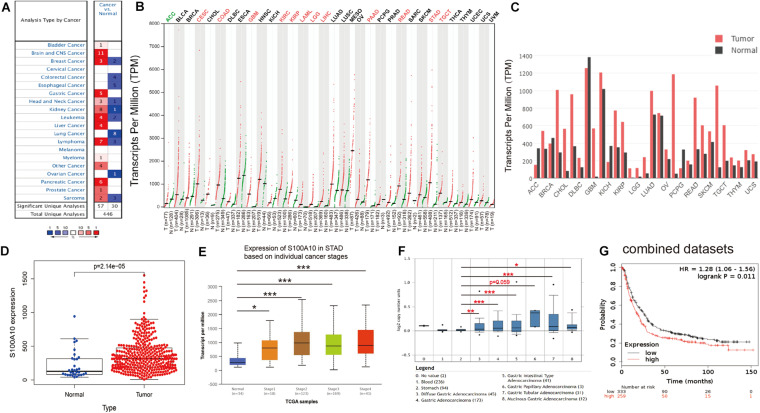
Evidence that S100A10 functions as an oncogene in GC. **(A)** The mRNA expression of S100A10 in different human cancers and corresponding normal tissues from the Oncomine database. The number in the colored cell is the number of datasets meeting the thresholds. Cell color is determined by the gene rank. The color intensity (red or blue) is directly proportional to the significance level of upregulation or downregulation, respectively. **(B,C)** S100A10 expression in cancers. Expression level of S100A10 across 33 TCGA tumors compared to TCGA normal and GTEx data using the GEPIA (Gene Expression Profiling Interactive Analysis) webserver. An obvious upregulation of this gene was evident in 12 cancers. For each TCGA tumor (red), its matched normal and GTEx data (green) are given. The abbreviations denote T: tumor; N: normal; n: number. Y-axis: transcript per million [log2 (TPM + 1)]. X-axis: number of tumor and normal samples. ACC: adrenocortical carcinoma; BLCA: bladder urothelial carcinoma; BRCA: breast invasive carcinoma; CESC: cervical squamous cell carcinoma and endocervical adenocarcinoma; CHOL: cholangiocarcinoma; COAD: colon adenocarcinoma; DLBC: lymphoid neoplasm diffuse large B-cell lymphoma; ESCA: esophageal carcinoma; GBM: glioblastoma multiforme; HNSC: head and neck squamous cell carcinoma; KICH: kidney chromophobe; KIRC: kidney renal clear cell carcinoma; KIRP: kidney renal papillary cell carcinoma; LAML: acute myeloid leukemia; LGG: brain lower grade glioma; LIHC: liver hepatocellular carcinoma; LUAD: lung adenocarcinoma; LUSC: lung squamous cell carcinoma; MESO: mesothelioma; OV: ovarian serous cystadenocarcinoma; PAAD: pancreatic adenocarcinoma; PCPG: pheochromocytoma and paraganglioma; PRAD: prostate adenocarcinoma; READ: rectum adenocarcinoma; SARC: sarcoma; SKCM: skin cutaneous melanoma; STAD: stomach adenocarcinoma; TGCT: testicular germ cell tumors; THCA: thyroid carcinoma; THYM: thymoma; UCEC: uterine corpus endometrial carcinoma; UCS: uterine carcinosarcoma; and UVM: uveal melanoma. **(D)** The expression level of S100A10 in GC and normal tissues using data from the TCGA database. **(E)** The expression of S100A10 in GC based on individual stages using the UALCAN database. **(F)** The expression of S100A10 in different GC subtypes compared to normal individuals derived from the Oncomine database. **(G)** S100A10 expression levels were associated with poor survival, as analyzed by the online program Kaplan-Meier Plotter. *P < 0.05, **P < 0.01, ***P < 0.001.

To assess the role of S100A10 in human carcinogenesis, S100A10 expression levels in various human cancers were analyzed using the Oncomine database. Compared to normal tissues, S100A10 was overexpressed in bladder cancer, brain cancer, breast cancer, GC, head and neck cancer, kidney cancer, leukemia, liver cancer, lymphoma, myeloma, pancreatic cancer, and prostate cancer (Figure 1A). To further determine the expression differences of S100A10 between tumor and normal tissues across multiple cancer types, the GEPIA database was utilized to confirm the mRNA expression of S100A10 in human cancer. S100A10 expression was significantly upregulated in cervical and endocervical cancers, colon adenocarcinoma, glioblastoma multiforme, kidney renal clear cell carcinoma, kidney renal papillary carcinoma, acute myeloid leukemia, brain lower grade glioma, liver hepatocellular carcinoma, pancreatic adenocarcinoma, rectum adenocarcinoma, stomach adenocarcinoma, and testicular germ cell tumors. The findings indicated the oncogene activity of S100A10 in malignant tumors of the digestive system (Figures 1B,C).

### S100A10 Is Upregulated in GC and Is Associated With Survival

Consistent with the results from the Oncomine and GEPIA databases, data from the TCGA database confirmed that the expression of S100A10 was elevated in GC samples compared to adjacent normal tissues (Figure 1D). Moreover, TCGA data analyzed by the UALCAN online tool confirmed that high expression of S100A10 was observed in Stage I-IV GC patients than normal controls (Figure 1E). In addition, data from the Oncomine database further demonstrated that higher expression of S100A10 was observed in diffuse gastric adenocarcinoma, gastric adenocarcinoma, gastric intestinal type adenocarcinoma, gastric papillary adenocarcinoma, gastric tubular adenocarcinoma, and mucinous gastric adenocarcinoma (Figure 1F). Concerning the prognostic value of S100A10, scrutiny of the Kaplan–Meier Plotter database indicated that a high level of S100A10 was closely associated with poor survival in GC patients (Figure 1G).

### S100A10 Expression Is Correlated With Tumor Glycolysis

**FIGURE 2:**
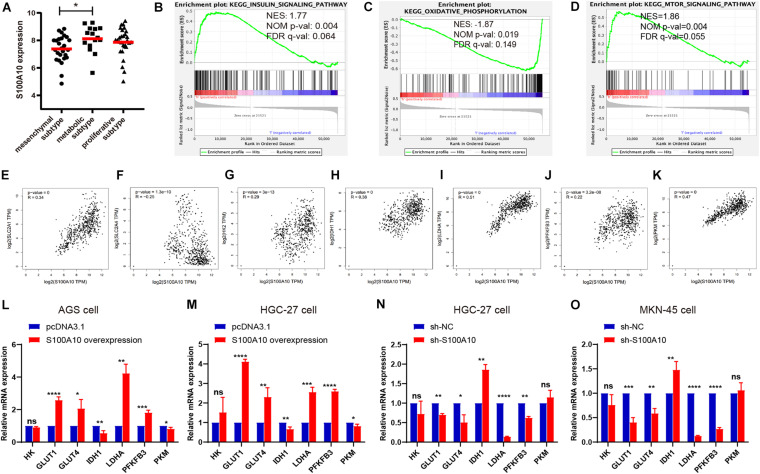
S100A10 is closely correlated with glycolysis. **(A)** S100A10 showed the highest expression in the metabolic subtype of GC. **(B–D)** RNA-seq data of stomach adenocarcinoma samples were downloaded from the TCGA database. These samples were defined as S100A10-high and S100A10-low groups based on S100A10 expression. Gene set enrichment analysis (GSEA) was then performed and revealed that S100A10 was closely related to insulin signaling pathway, oxidative phosphorylation and mTOR signaling pathway. NES, normalized enrichment score; NOM, nominal; FDR, false discovery rate. **(E–K)** Correlation analysis between S100A10 and glycolytic transporters or enzymes in GC, including SLC2A1 (GLUT1), SLC2A4 (GLUT4), HK2, IDH1, LDHA, PFKFB3, and PKM. **(L–O)** The mRNA expression levels of glycolytic transporters or enzymes were assessed by RT-qPCR after S100A10 overexpression or knockdown. *P < 0.05, **P < 0.01, ***P < 0.001.

To determine the potential oncogenic effect of S100A10 in GC, we examined the normalized data from GEO datasets GSE35809. S100A10 showed maximum expression in the metabolic subtype (Figure 2A). GSEA was utilized to analyze GC tissue samples using the RNA-seq data from the TCGA database. Based on S100A10 expression, the samples were defined as a S100A10-high group (top 50%) and a S100A10-low group (bottom 50%). The GSEA analysis revealed that the top enriched pathways were energy-related. Gene sets related to the insulin signaling and mTOR signaling pathways were enriched in the S100A10-high group. Gene sets related to oxidative phosphorylation were differentially enriched in the S100A10-low group (Figures 2B–D). The involvement of these three signaling pathways in glycolysis has been described (Yuan et al., 2016).

There was no evidence of S100A10 involvement in cancer glycolysis. Therefore, we aimed to explore the role of S100A10 in GC glycolysis. A correlation analysis was performed between S100A10 and glycolytic components, such as glucose transporter (SLC2A1 and SLC2A2), glycolytic enzymes (HK2, PFKFB3, and PKM), the key regulatory enzyme for the tricarboxylic acid (TCA) cycle (IDH1), and lactate generation catalytic enzyme (LDHA), using data from the GEPIA database. All the glycolytic components analyzed were closely related to S100A10 (Figures 2E–K), indicating that S100A10 might be involved in glycolytic metabolism in GC.

We next asked whether S100A10 promotes glycolysis in GC. First, the protein expression levels of S100A10 in GC cells were verified by western blotting (Supplementary Figures S1A,B). According to the protein expression level, cell lines were categorized as high (MKN-45), intermediate (HGC-27), and low (AGS) S100A10-expressing cell lines. Thus, we established the overexpression of S100A10 in AGS and HGC-27 cells by transfection with S100A10-overexpression plasmid, and conducted S100A10 knockdown in MKN-45 and HGC-27 cells through transfection with si-RNA (si-S100A10) or S100A10-silencing lentivirus (sh-S100A10). The expression levels of S100A10 in the resultant cell lines were also verified by RT-qPCR and western blotting (Supplementary Figures S1C–I).

RT-qPCR was performed to investigate the changes in glycolytic components affected by S100A10. Consistently, S100A10-overexpressing cells exhibited increased GLUT1, GLUT4, LDHA, and PFKFB3 mRNA expression levels and decreased IDH1 mRNA expression levels, while S100A10 knockdown produced the opposite trend. Additionally, S100A10 had no remarkable effects on HK and PKM expression (Figures 2L–O). Collectively, these results indicated that S100A10 promotes aerobic glycolysis in GC.

### S100A10 Favors Glucose Consumption Through GLUT1 in GC

**FIGURE 3:**
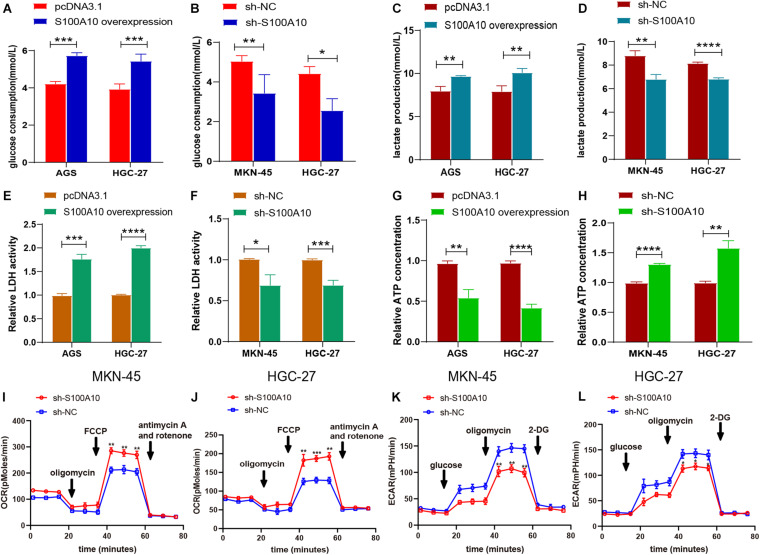
S100A10 promotes aerobic glycolysis in GC. **(A–H)** The influence of S100A10 overexpression or knockdown on glucose consumption **(A,B)**, lactate production **(C,D)**, relative LDH activity **(E,F)**, and ATP production **(G,H)** in GC cells. **(I–L)** The influence of S100A10 knockdown on the extracellular acidification ratio (ECAR) and oxygen consumption ratio (OCR) in GC cells. *P < 0.05, **P < 0.01, ***P < 0.001.

Elevated glucose consumption, which leads to aerobic glycolysis, is considered an evolutionary advantage for cancer cells (Yuan et al., 2016). Glucose consumption was measured using a glucose assay kit. S100A10-overexpressing cells displayed significantly increased glucose consumption compared with the negative control, while glucose consumption was reduced in S100A10-knockdown cells (Figures 3A,B). Increasing evidence has shown that the family of glucose transporters (GLUTs) plays important roles in cancer glucose metabolism and cancer progression (Yuan et al., 2016). Glucose transporters, in particular SLC2A1 (GLUT1), are a key rate-limiting factor in glucose metabolism in cancer cells (Jozwiak and Lipinska, 2012). Presently, there was a notable increase in the mRNA and protein expression levels of GLUT1 in S100A10-overexpressing GC cells. The opposite effect was observed following S100A10 knockdown (Figures 2L–O, 5A). The findings indicated that S100A10 promotes glucose consumption in GC as mediated by GLUT1 upregulation.

### S100A10 Facilitates Lactate Production by Modulating Glycolytic Enzymes

Aerobic glycolysis markedly increases the lactate concentration. This produces an acidic microenvironment and drives cancer progression. A lactate assay kit was used to measure the production of lactate in GC. As shown in Figure 3C, S100A10 overexpression enhanced lactate generation in AGS and HGC-27 cells. Lactate production was significantly decreased in S100A10-knockdown cells (Figure 3D). The enzymes regulating glycolytic flux during glucose metabolism (LDHA and PFKFB3) were chosen for further investigation. LDHA is an important regulator of lactate production in aerobic glycolysis (Liu et al., 2015). PFKFB3, the key rate-limiting enzyme, leads to enhanced glycolysis, which is essential for tumor cell survival (Han et al., 2017). The mRNA and protein expression levels of LDHA and PFKFB3, and the enzymatic activity of LDH were evaluated in S100A10-overexpressing and S100A10-knockdown cells. S100A10 overexpression remarkably upregulated the enzymatic activity of LDH and the expressions of LDHA and PFKFB3, whereas inhibition of S100A10 expression attenuated these events (Figures 2L–O, 3E,F, 5A). The findings suggested that S100A10 can upregulate the activities of key glycolytic enzymes to enhance lactate production in GC.

### S100A10 Reduces ATP Production by Suppressing Oxidative Phosphorylation in GC

Most cancer cells are more likely to adopt a less efficient aerobic glycolysis for energy supply, which is critical for their rapid proliferation. Cellular ATP levels were measured using an ATP assay kit. As shown in Figures 3G,H, S100A10 overexpression reduced intracellular ATP production, while S100A10 depletion increased ATP synthesis. These findings indicated that S100A10 increased glycolysis resulted in decreased ATP production. To further investigate S100A10 modulation in the glucose metabolic process, ECAR and OCR were measured. OCR was significantly increased in S100A10-silencing cells (Figures 3I,J), indicating that S100A10 knockdown increased ATP production by recovering oxidative phosphorylation. ECAR was markedly decreased after S100A10 knockdown (Figures 3K,L), suggesting that S100A10 knockdown could effectively suppress aerobic glycolysis. Overall, these results indicated that S100A10 knockdown increased ATP production by remodeling glucose metabolism from aerobic glycolysis to mitochondrial oxidative phosphorylation.

### S100A10 Promotes Malignant Proliferation in GC

**FIGURE 4:**
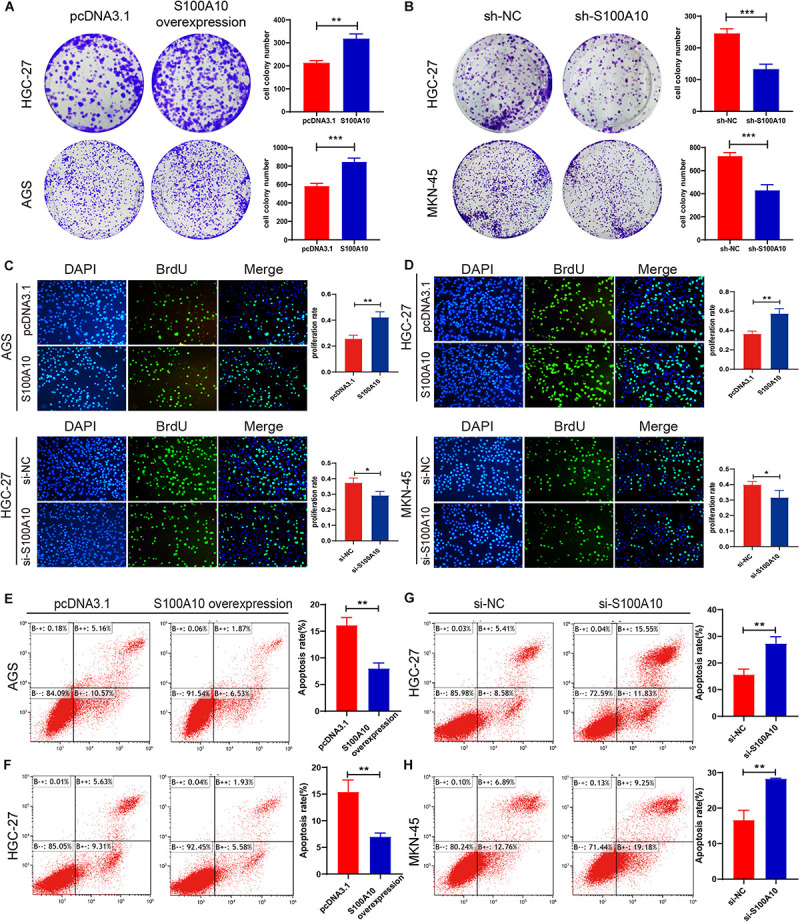
S100A10 promotes cell proliferation and inhibits apoptosis in GC cells. **(A–D)** Colony formation assay and BrdU assay demonstrated that increased S100A10 expression promoted cell proliferation and DNA synthesis capacity. **(A,C)**, while knockdown of S100A10 inhibited cell growth **(B,D)**. **(E–H)** Increased S100A10 inhibited cell apoptosis **(E,F)**, and silencing S100A10 promoted cell apoptosis **(G,H)**. *P < 0.05, **P < 0.01, ***P < 0.001.

The effect of S100A10 on GC cell growth was examined by plate colony formation and BrdU assay in GC cells. Overexpression of S100A10 dramatically promoted GC cell proliferation and DNA synthesis activities, whereas knockdown of S100A10 significantly suppressed cell rapid growth (Figures 4A–D). The collective results indicated that S100A10 could enhance the proliferation of GC cells.

**FIGURE 5:**
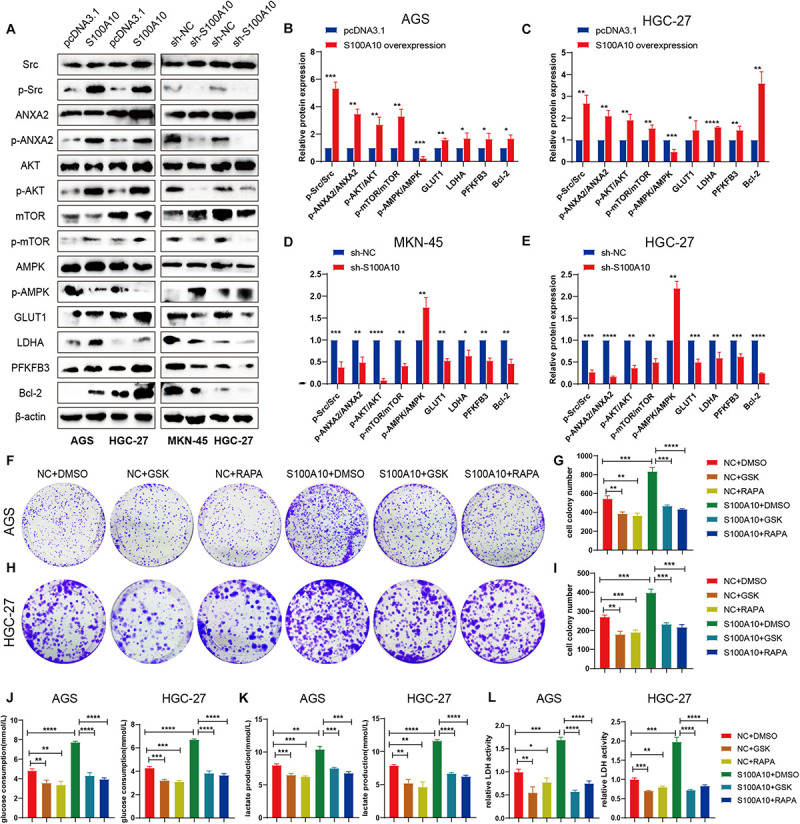
S100A10 activates the mTOR signaling pathway to promote glycolysis process. **(A–E)** After S100A10 expression was overexpressed or down regulated, the molecules of downstream signaling pathway were measured by western blot analysis. **(F–I)** The mTOR inhibitor, rapamycin (RAPA), and the LDHA inhibitor GSK-2837808A (GSK), effectively abolished the promoting effects of S100A10 on cell proliferation. **(J–L)** Rapamycin and GSK-2837808A could significantly rescue the promoting effect of S10A10 on glucose consumption, lactate production and LDH activity.

### S100A10 Suppresses Apoptosis of GC Cells

The role of S100A10 in cell apoptosis was evaluated by flow cytometry. High S100A10 expression decreased the rate of apoptosis (Figures 4E,F), while the apoptosis rate was obviously enhanced after interfering with S100A10 (Figures 4G,H). These results indicated the involvement of S100A10 in GC cell apoptosis. Expression of the anti-apoptotic protein Bcl-2 was significantly upregulated by S100A10 overexpression in both AGS and HGC-27 cells, while silencing of the expression of S100A10 was accompanied by downregulation of Bcl-2 expression (Figure 5A). Collectively, these results showed that S100A10 inhibited cell apoptosis in GC by regulating Bcl-2 expression.

### S100A10 Regulates GC Cells Glycolysis by Activating the mTOR Pathway

The results of GSEA using the TCGA database demonstrated that the mTOR signaling pathway was closely correlated with S100A10 expression. Previous studies reported that S100A10 is usually bound to its ligand ANXA2 to form the S100A10-ANXA2 complex (Noye et al., 2018). High expression of S100A10 is essential for ANXA2 phosphorylation mediated by Src kinase in leukemia (Spijkers-Hagelstein et al., 2013). Furthermore, phosphorylated ANXA2 can further activate the AKT/mTOR signaling pathway to regulate cell proliferation (Kagawa et al., 2012; Zhang et al., 2018). Therefore, we hypothesized that the high expression of S100A10 may activate Src kinase and promote the phosphorylation of Annexin A2, which in turn can activate the AKT/mTOR signaling pathway.

Western blot analysis was performed. The phospho-Src (p-Src), phospho-ANXA2 (p-ANXA2), phospho-AKT (p-AKT) and phospho-mTOR (p-mTOR) expression levels were normalized to total Src, ANXA2, AKT and mTOR (p-Src/Src, p-ANXA2/ANXA2, p-AKT/AKT, p-mTOR/mTOR). Presently, the p-Src/Src, p-ANXA2/ANXA2, p-AKT/AKT, p-mTOR/mTOR ratios were increased in both AGS and HGC-27 cells after S100A10 overexpression (Figure 5A), suggesting that S100A10 functions by activating the Src/ANXA2/AKT/mTOR signaling pathway. In addition, changes in mitochondrial respiration induced by ectopic S100A10 expression reduced ATP production, followed by AMPK inactivation. The expression level of mTOR downstream molecules related to glycolysis, such as GLUT1, PFKFB3, and LDHA, were notably elevated in S100A10-overexpressing cells. The anti-apoptotic protein Bcl-2 was upregulated along with S100A10 overexpression. The opposite finding was observed following the inhibition of S100A10 expression (Figures 5A–E). These findings, combined with the aforementioned metabolic alterations, demonstrated that S100A10 induced glycolysis by activating the Src/ANXA2/AKT/mTOR signaling pathway, leading to aerobic glycolysis, apoptotic resistance, and rapid proliferation of GC cells.

### S100A10 Mediated Promotion Can Be Abolished by mTOR and LDHA Inhibitors

Given the molecular association of S100A10 with the mTOR pathway, we next explored whether mTOR mediates the facilitative effects of S100A10 on GC cell proliferation and glycolysis. A rescue experiment was performed to confirm that the inhibition of the mTOR pathway could abolish the promotion of glycolysis and the malignant phenotype of GC cells induced by ectopic S100A10 expression. In the experiment, we abolished the activity of mTOR with rapamycin (RAPA) and inhibited LDHA using GSK-2837808A (GSK) in GC cells. Glycolytic activity was examined. Rapamycin and GSK could significantly decrease cancer cell proliferation, glucose consumption, lactate production and LDH activity. As expected, rapamycin and GSK completely abrogated the effects of S100A10 overexpression on colony formation and aerobic glycolysis (Figures 5F–L). Taken together, these results demonstrated that S100A10 promotes aerobic glycolysis and cell proliferation in GC cells through the mTOR pathway.

### S100A10 Promotes Tumor Growth in a Xenograft Mouse Model

**FIGURE 6:**
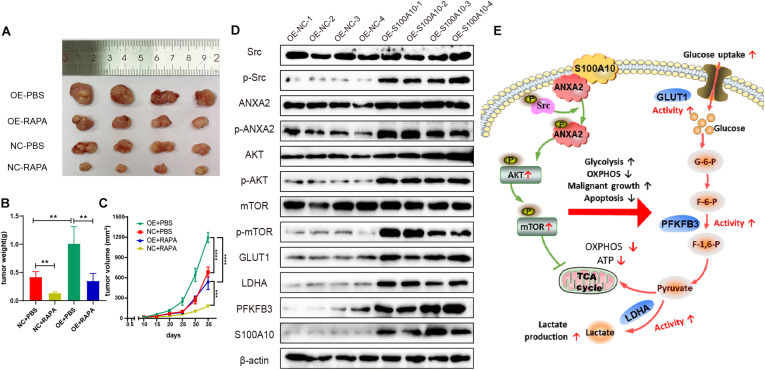
S100A10 promotes tumor growth *in vivo*. **(A–C)** S100A10 overexpression promoted tumor growth *in vivo*. This effect could be rescued by rapamycin. **(D)** Expression of downstream effector genes of S100A10 was evaluated in mouse xenograft tumors. The expression levels of p-Src, p-ANXA2, p-mTOR, GLUT1, LDHA, and PFKFB3 were significantly higher in the OE-S100A10 group compared with negative control group. **(E)** A schematic model of the role and mechanism of S100A10 in GC glycolysis. Ectopic S100A10 expression increases Src kinase activity, resulting in the phosphorylation of S100A10 binding protein, ANXA2, which in turn activates the AKT/mTOR signaling pathway and further upregulates the activity of key glycolytic enzymes to enhance aerobic glycolysis and malignant growth and suppress cell apoptosis in GC. OXPHOS, oxidative phosphorylation; TCA cycle, tricarboxylic acid cycle; and ATP, adenosine triphosphate.

To further investigate the role of S100A10 in driving tumorigenesis *in vivo*, a subcutaneous tumor model was established in nude mice. First, we established HGC-27 cells that stably overexpressed S100A10 (OE-S100A10) and the corresponding control cells (OE-NC). After lentiviral infection, all HGC-27 cells expressed green fluorescent protein (GFP), suggesting successful cell infection (Supplementary Figure S1J). S100A10 overexpression was confirmed by western blotting (Supplementary Figure S1K–L). The stably transfected GC cells were subcutaneously inoculated into nude mice. The mice were subsequently treated with PBS or rapamycin. As shown in Figures 6A–C, the OE-S100A10 group demonstrated a much larger average tumor volume and tumor weight compared with the OE-NC group. Moreover, rapamycin treatment further inhibited the promoting effect of S100A10 on tumor growth (Figures 6A–C).

In addition, expression of downstream effector genes were evaluated in mouse xenograft tumors. The expression levels of p-Src, p-ANXA2, p-mTOR, GLUT1, LDHA, and PFKFB3 were significantly higher in the OE-S100A10 group compared with negative control group (Figure 6D). These collective results indicated the importance of S100A10 in promoting GC growth through the mTOR pathway. The results also showed that the growth promoting effects were markedly abated by rapamycin.

## Discussion

In the current study, we demonstrate that S100A10 promotes GC aerobic glycolysis and cell proliferation and suppresses cell apoptosis. Our findings indicate that S100A10 plays an oncogenic role in GC. The results provide the first evidence that S100A10 drives tumorigenesis via the mTOR signaling pathway. The findings increase the knowledge of the biological functions of S100A10 in tumorigenesis.

Previous studies have demonstrated the crucial role of S100A10 in the malignant progression of GC (Wang C. et al., 2019). The results also showed that S100A10 promoted malignant growth in GC *in vitro* and *in vivo*. To achieve rapid proliferation, cancer cells adjust their energy source from oxidative phosphorylation to aerobic glycolysis. GSEA results indicated a potential role for S100A10 in glycolysis. Elevated glucose consumption and lactate production were observed in S100A10-overexpressed GC cells, verifying that S100A10 plays an important role in the facilitation of glycolysis. S100A10 knockdown significantly blocked glucose metabolism and induced a switch from aerobic glycolysis to mitochondrial respiration. To the best of our knowledge, the present study is the first to suggest that S100A10 is a critical mediator involved in aerobic glycolysis and that S100A10 knockdown has the potential to inhibit glycolysis.

Increasing evidence has indicated the close link between glycolysis and resistance to apoptosis in tumor progression and poor patient outcomes (Otsuki et al., 2005; Gu et al., 2017). Cancer cells manipulate metabolic regulation to escape apoptosis and cell death (Matsuura et al., 2016). No studies have explored the biological characteristics of S100A10 in GC apoptosis. The data of the current study suggested that ectopic S100A10 expression could significantly reduce apoptosis. GC cells overexpressing S100A10 displayed significantly elevated expression of Bcl-2, an important anti-apoptotic protein. We conclude that the ectopic expression of S100A10 alters glycolysis metabolism to promote apoptosis resistance. This may allow cancer cells to survive the harsh tumor microenvironment.

The high expression of S100A10 activated Src kinase and promoted the phosphorylation of ANXA2, which in turn activated the AKT/mTOR signaling pathway. Prior studies have shown that the mTOR pathway contributes to various cellular processes that include cell proliferation, survival, autophagy, and aerobic glycolytic metabolism (Francipane and Lagasse, 2014; Kim and Guan, 2015; Morita et al., 2015). Of note, the GSEA results revealed a positive correlation between S100A10 expression and mTOR signaling pathways. We observed that p-mTOR levels were greatly enhanced in S100A10 overexpressing GC cells as well as the increased levels of downstream targets of mTOR, such as LDHA, PFKFB3, GLUT1, and Bcl-2. These proteins directly control cancer cell glucose metabolism, proliferation, and apoptosis. Moreover, treatment with an mTOR inhibitor or LDHA inhibitor could abrogate the promoting effects of S100A10 on aerobic glycolysis and cell proliferation, suggesting that S100A10 is a key mediator of the metabolic process through the mTOR signaling pathway. An inhibitor of mTOR is useful for the treatment of GC patients with high S100A10 expression.

Aerobic glycolysis facilitates tumor progression by accelerating cancer cells growth. Molecular targeted therapy has been a key advance in the control of tumor growth and in the development of novel anticancer treatments. Glycolysis consists of multiple steps that require several essential glycolytic enzymes. For example, GLUT is a key rate-limiting factor in the transport and metabolism of glucose in cancer cells. GLUT enhances glucose uptake to support rapid proliferation (Jozwiak and Lipinska, 2012). PFKFB3 is a key glycolysis regulator that has been proposed to act as a tumor promoter by accentuating cell migration and invasion (Han et al., 2017). LDHA catalyzes the conversion of pyruvate to lactate. The enzyme is a crucial mediator of aerobic glycolysis (Liu et al., 2015). Interfering with the key enzymes of aerobic glycolysis in cancer cells to block glucose metabolism may provide a novel and promising therapeutic strategy for GC. However, focusing on a single target is considered to have limited impact because of tumor heterogeneity. In this study, silencing the expression of S100A10 could effectively inhibit the activity of key enzymes in the glycolysis-related pathways, which makes it a far more attractive target than the single target approach. S100A10 could be an excellent target for cancer treatment. Diverse therapeutic strategies that have been used to target S100A10 include neutralizing antibodies, small molecule inhibitors, peptides, and all−trans retinoic acid (ATRA) (Bresnick, 2018; Saiki and Horii, 2019). S100A10 antibody plays an important role in reducing homing of leukemia cells to the bone marrow *in vivo* (Gopalakrishnapillai et al., 2015). Treatment of leukemic cells with ATRA could effectively induce S100A10 degradation. Considering the link between the molecular biology of S100A10 and GC, further studies are needed to determine whether targeting S100A10 therapeutic intervention inhibits carcinogenesis.

## Conclusion

In conclusion, the present study provides the first evidence that S100A10 may function as a positive regulator of glycolysis in GC. Knockdown of S100A10 expression suppresses the activation of the mTOR pathway, thus inhibiting glycolysis and tumor growth. S100A10 could be a potential therapeutic target for the treatment of GC in the future.

## Data Availability Statement

The datasets presented in this study can be found in online repositories. The names of the repository/repositories and accession number(s) can be found in the article/Supplementary Material.

## Ethics Statement

The animal study was reviewed and approved by Ethical Committee on Scientific Research of Shandong University Qilu Hospital.

## Author Contributions

YL, X-YL, R-CZ, XG, B-YJ, and BL performed all experiments. YL, L-XL, and YS performed data analysis. YL wrote the manuscript. X-LZ, Y-QL, and YL designed the overall project and revised the manuscript. All authors have read and approved the final manuscript.

## Conflict of Interest

The authors declare that the research was conducted in the absence of any commercial or financial relationships that could be construed as a potential conflict of interest.

## Footnotes

1 http://www.oncomine.org

2 http://gepia.cancer-pku.cn/

3 http://cancergenome.nih.gov/

4 http://ualcan.path.uab.edu

5 http://www.ncbi.nlm.nih.gov/geo
