# Supplementary material for: S100A10 Accelerates Aerobic Glycolysis and Malignant Growth by Activating mTOR-Signaling Pathway in Gastric Cancer
Source: Front Cell Dev Biol. 2020 Nov 26;8:559486. doi: 10.3389/fcell.2020.559486 (PMC7726224; doi:10.3389/fcell.2020.559486)
Supplement: Supplementary file 1 [file Data_Sheet_1.docx]

| **Supplementary Table S1. qPCR primers sequences** | |
| --- | --- |
| **Definition** | **Sequence** |
| *GAPDH*-F | GCACCGTCAAGGCTGAGAAC |
| *GAPDH*-R | TGGTGAAGACGCCAGTGGA |
| *S100A10*-F | AACAAAGGAGGACCTGAGAGTAC |
| *S100A10*-R | CTTTGCCATCTCTACACTGGTCC |
| *GLUT1*-F | TGTGGGCATGTGCTTCCAGTA |
| *GLUT1*-R | CGGCCTTTAGTCTCAGGAACTTTG |
| *GLUT4*-F | GGGCTGAGACAGGGACCATAAC |
| *GLUT4*-R | CATGAGCAATGGCATCCAGAA |
| *HK2*-F | GAGTTTGACCTGGATGTGGTTGC |
| *HK2-R* | CCTCCATGTAGCAGGCATTGCT |
| *IDH1*-F | AATCAGTGGCGGTTCTGTGGTA |
| *IDH1*-R | ACTTGGTCGTTGGTGGCATC |
| *LDHA-F* | GGATCTCCAACATGGCAGCCTT |
| *LDHA-R* | AGACGGCTTTCTCCCTCTTGCT |
| *PKM-F* | ATGGCTGACACATTCCTGGAGC |
| *PKM-R* | CCTTCAACGTCTCCACTGATCG |
| *PFKFB3-F* | GGCAGGAGAATGTGCTGGTCAT |
| *PFKFB3-2* | CATAAGCGACAGGCGTCAGTTTC |


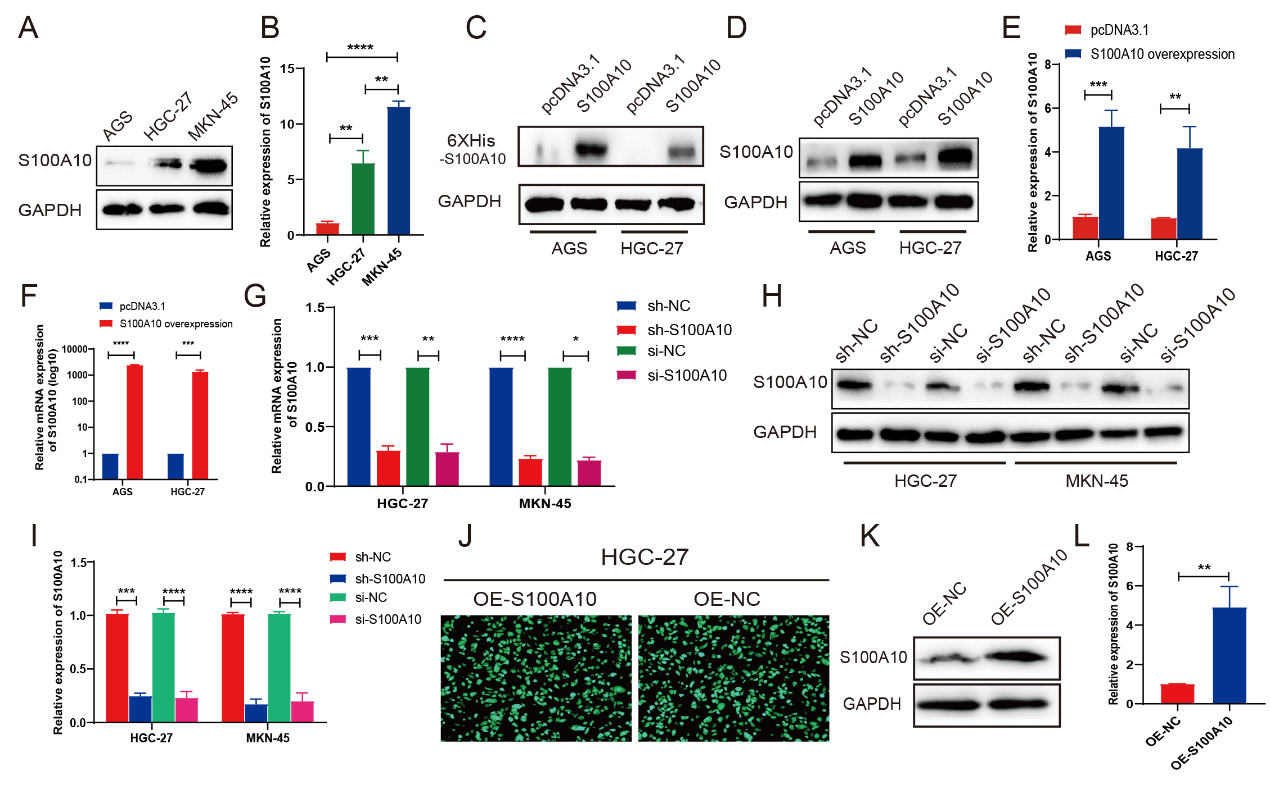


**Supplementary Figure S1. The overexpression and knock‐down efficiency of S100A10 in gastric cancer cells**.

(A and B) The expression of S100A10 in gastric cancer cell lines, AGS, HGC-27 and MKN-45. (C-E) Overexpression efficiency of S100A10 induced by S100A10-overexpressed plasmid in AGS and HGC-27 cells. (F-G) The mRNA expression levels of S100A10 in gastric cancer cells after S100A10 overexpression or knockdown. (H and I) The protein expression levels of S100A10 in gastric cancer cells after S100A10 knockdown (J) The infection efficiency of lentivirus in HGC-27 cells was confirmed by fluorescence microscopy. (K and L) Overexpression efficiency of S100A10 induced by S100A10-overexpressed lentivirus in HGC-27 cells.
